# A 12.3-kb Duplication Within the *VWF* Gene in Pigs Affected by Von Willebrand Disease Type 3

**DOI:** 10.1534/g3.117.300432

**Published:** 2017-12-05

**Authors:** Stefanie Lehner, Mahnaz Ekhlasi-Hundrieser, Carsten Detering, Hanna Allerkamp, Christiane Pfarrer, Mario von Depka Prondzinski

**Affiliations:** * Department of Fundamental Research, Werlhof Institute GmbH, 30159 Hannover, Germany; † Institute for Anatomy, University of Veterinary Medicine Hannover Foundation, 30173 Hannover, Germany

**Keywords:** *Sus scrofa*, Von Willebrand factor, Bleeding disorder, Frameshift, Nonsense-mediated decay

## Abstract

Von Willebrand Disease (VWD) type 3 is a serious and sometimes fatal hereditary bleeding disorder. In pigs, the disease has been known for decades, and affected animals are used as models for the human disease. Due to the recessive mode of inheritance of VWD type 3, severe bleeding is typically seen in homozygous individuals. We sequenced the complete porcine *VWF* (Von Willebrand Factor) complementary DNA (cDNA) and detected a tandem duplication of exons 17 and 18, causing a frameshift and a premature termination codon (p.Val814LeufsTer3) in the affected pig. Subsequent next generation sequencing on genomic DNA proved the existence of a 12.3-kb tandem duplication associated with VWD. This duplication putatively originates from porcine Short Interspersed Nuclear Elements (SINEs) located within *VWF* introns 16 and 18 with high identity. The premature termination truncates the *VWF* open reading frame by a large part, resulting in an almost entire loss of the mature peptide. It is therefore supposed to account for the severe VWD type 3. Our results further indicate the presence of strong, nonsense-mediated decay in *VWF* messenger RNA (mRNA) containing the duplication, which was supported by the almost complete absence of the complete VWF protein in immunohistochemistry analysis of the VWD-affected pig. In the past, differentiation of wild-type and heterozygous pigs in this VWD colony had to rely on clinical examinations and additional laboratory methods. The present study provides the basis to distinguish both genotypes by performing a rapid and simple genetic analysis.

Von Willebrand disease (VWD) is a common bleeding disorder known in human and many animal species. It is differentiated into three types, where type 1 comprises partial quantitative deficiencies of the Von Willebrand factor (VWF) protein, type 2 comprises several qualitative disorders, and type 3 a virtually complete quantitative deficiency of the functional protein (Castaman *et al.* 2013; Sadler *et al.* 2006). In the majority of cases, VWD is caused by genetic variants within the *VWF* gene. VWD type 3 usually causes the most severe clinical signs with excessive mucocutaneous as well as muscle and joint bleeding (Metjian *et al.* 2009). Plasma VWF is absent or distinctly reduced with concentrations below 5 IU/dl and factor VIII (FVIII) clotting activity is significantly reduced (Goodeve and James 2014). Without treatment by repeated infusions of VWF/FVIII clotting factor concentrate, affected individuals may also bleed to death. VWD type 3 is the least common, accounting for <5% of all VWD cases, and shows a recessive mode of inheritance (Goodeve and James 2014).

Besides human, causal variants for VWD type 3 have been reported for different dog breeds. In Dutch Kooiker dogs, the disease is caused by a base substitution within intron 16 of *VWF* affecting a splice site and thus resulting in a frameshift and a premature stop codon (Rieger *et al.* 1998). Furthermore, a deletion of 1 base within *VWF* exon 4 was detected in affected Scottish Terriers (Venta *et al.* 2000) and another 1-base deletion within exon 7 is the putative cause for VWD in the Shetland Sheepdog (Venta *et al.* 1998). VWD type 3 was also diagnosed in a Himalayan cat (French *et al.* 1987), but the causal genetic background was not analyzed.

Pigs affected by a “hemophilia-like bleeding disorder” were first reported in 1941 (Hogan *et al.* 1941), and the disease was defined as a recessively inherited form of VWD some decades later (Bowie *et al.* 1973; Griggs *et al.* 1974). VWD-affected pigs showed severe bleeding and had no detectable platelet aggregation activity, while carriers for the disease showed reduced platelet aggregation levels of ∼50% and no bleeding (Griggs *et al.* 1974). They are phenotypically identical to VWD type 3 in humans (Brouland *et al.* 1999) and therefore are a valuable large animal model for the human disease. VWD in pigs has been localized at the *VWF* locus (Bahou *et al.* 1988), but the molecular mechanism and the causal genetic background remained undetected.

As for our further research on VWD the precise genetic background as well as genotype determination was required, we sequenced the complete *VWF* complementary DNA (cDNA) as well as parts of the genomic DNA in pigs of each genotype to identify the putative causal genetic variant.

## Materials and Methods

### Animals and sampling

Samples of the pigs used were purchased from Hôpital Lariboisière, Paris, France. All animal experiments were performed according to the local regulations. The protocol involving animal husbandry and killing of the pigs was approved by the French Ethical Committee for Animal Experimentation and the French Ministry of Research, Department of Animal Experimentation and Project Authorization (# 0130001).

The pigs were bred in France and trace back to the original VWD colony of the Mayo Clinic (Dr. E. J. W. Bowie, Rochester, MN). The original population of VWD-affected pigs was of Poland China breed and has been cross-bred with Yorkshire-Hampshire and Meishan pigs. Further specific characteristics of these pigs and their phenotype have been elaborately described (Bowie *et al.* 1973; Fass *et al.* 1979; Roussi *et al.* 1998).

The phenotype of each pig was assigned by clinical examination as well as determination of the Von Willebrand factor antigen blood plasma level (VWF:AG) using the STA-Liatest VWF:Ag test kit (Diagnostica Stago S.A.S, Asnières sur Seine, France) and a specific calibration curve for porcine plasma. We considered the VWD-affected phenotype in a pig safely assigned if clinical signs were present and VWF:AG < 3% (Roussi *et al.* 1996). VWD-carrier pigs were reported to show VWF:AG values of 42 ± 11% while values in VWD-unaffected pigs were measured at 96.6 ± 22.7% (Roussi *et al.* 1996). However, humans unaffected by VWD type 3 may show values of VWF:AG above 50% (Goodeve and James 2014), while carriers for VWD type 3 may show values of VWF:AG between 20 and 80% (Gadisseur *et al.* 2013). Therefore, we considered the phenotype secured in our pigs if no or mild clinical signs were present and VWF:AG was between 20 and 50% (VWD-carrier) or if no clinical signs were present and VWF:AG > 80% (VWD-unaffected).

Uterus and esophagus tissue samples of five pigs (*Sus scrofa*) were taken directly after death of the animals and stored in RNA*later* RNA-stabilizing solution or embedded in paraffin wax. The pigs had been born to different litters. One of these pigs was affected by VWD type 3 (VWF:AG < 3%), two were VWD-carriers (VWF:AG 20 and 34%, respectively), and two were VWD-unaffected controls (VWF:AG 103 and 112%, respectively). The pig affected by VWD showed a severely prolonged bleeding time.

In addition, hair samples of 32 pigs were used. In 28 of these, the VWD phenotype had been confidently assigned. Of these, 12 pigs were VWD-affected, 12 were VWD-carriers, and four were VWD-unaffected. For the remaining four pigs, the VWF:AG values were unknown. However, these animals originated from pig lines in which VWD had never been diagnosed before and were not related to such lines. Therefore, they were considered VWD-unaffected.

### Isolation of nucleic acid

RNA was isolated from uterus tissue samples using the Maxwell 16 Tissue LEV Total RNA Purification Kit (Promega GmbH, Mannheim, Germany) according to the manufacturer’s instructions. The reverse transcription of messenger RNA (mRNA) into cDNA was performed using the GoScript Reverse Transcription System (Promega GmbH) according to the technical manual.

For extraction of genomic DNA from uterus tissue samples, we used the Maxwell 16 LEV Blood DNA Kit (Promega GmbH) with a modified lysis protocol (crushing a piece of tissue of ∼5 mm in diameter, incubation at 56° for 2 hr with 400 µl lysis buffer and 40 µl proteinase K). Extraction of genomic DNA from hair roots was also performed using the Maxwell 16 LEV Blood DNA Kit (Promega GmbH) and the same modified lysis protocol as described above, omitting the crushing step.

### Sanger sequencing

Primers were designed using the Primer3 software version 4.0.0 (Koressaar and Remm 2007) to generate 17 products spanning the complete porcine *VWF* cDNA and one additional product that provided a better view at the exon 18–exon 19 connection, as well as two products of genomic DNA of exons 18 and 37 (Supplemental Material, Table S1). To determine both break points of the duplication as well as the duplication junction on genomic DNA, we designed three additional pairs of primers (Table S1).

Sequencing reactions of these PCR products were performed using the GenomeLab DTCS Quick Start Kit (Beckman Coulter, Brea, CA). Products were purified by Agencourt Clean Seq (Beckman Coulter) and directly sequenced on a CEQ 8800 Genetic Analysis System (Beckman Coulter). Chromatograms were evaluated using the CEQuence Investigator Module (Beckman Coulter) and the Chromas software version 2.4.4 (http://technelysium.com.au). All variants causing an amino acid exchange were analyzed for potential functional effects using the PolyPhen-2 software (Adzhubei *et al.* 2010). Sequences flanking the duplication break points within intron 16 and intron 18 were evaluated using RepeatMasker Web Server 4.0.5 (http://www.repeatmasker.org/) and NCBI Align Sequences Nucleotide BLAST (bl2seq; http://blast.ncbi.nlm.nih.gov/).

### Next generation sequencing

To capture the region including the duplication junction, we applied long-range PCR using the GoTaq Long PCR Master Mix (Promega GmbH) as well as a forward primer located in exon 18 and a reverse primer located in exon 17, giving a 10,214-bp product in animals homozygous or heterozygous for the duplication (Table S1). Primers were designed as described above. Genomic DNA of the three different pigs heterozygous or homozygous for VWD was amplified in duplicates. Amplicon libraries for NGS of this product were prepared using the Nextera XT DNA Library Preparation Kit (Illumina, San Diego, CA) according to the manufacturer’s instructions. All DNA samples were individually indexed during the hybridization step previously to library PCR amplification. Purification of PCR products was performed using Exonuclease I and Shrimp Alkaline Phosphatase and concentration of double-stranded product DNA was determined using the QuantiFluor double-stranded DNA (dsDNA) system (Promega GmbH). Concentration of the product was adjusted to 0.2 ng/µl. The sample library was loaded onto a MiSeq Sequencing System (Illumina), according to the manufacturer’s instructions using the MiSeq Reagent Kit V2. Sequences were assembled *de novo* using the SPAdes Genome Assembler version 3.9.0 (Nurk *et al.* 2013) after adapter trimming and quality filtering using BBduk (BBMAP version 36.20; http://sourceforge.net/projects/bbmap/). Subsequently, the reads of each sample were mapped to the *de novo* assembled sequence (GenBank accession number: MG372112) using BWA (Li and Durbin 2009). Duplicates were removed using Picard (http://broadinstitute.github.io/picard/). Both procedures were performed using the Galaxy web platform (usegalaxy.org; Afgan *et al.* 2016). Due to the short size of the sequenced product of 10.2 kb, the resulting average sequencing depth was as high as 18,183 on average and ranged from 16,665 to 19,618 between the samples.

### Immunohistochemistry

Esophagus tissue samples of five pigs were embedded in paraffin wax and sectioned (3 µm) using a rotary microtome (Leitz, Wetzlar, Germany). After production of histological sections, the expression of VWF (Polyclonal Rabbit Anti-Human Von Willebrand Factor antibody, A0082, dilution 1:3000; Dako, Glostrup, Denmark) was determined by immunohistochemistry according to the working groups standard procedures (Haeger *et al.* 2015). The endothelium of all samples was investigated for staining intensity.

### Genetic testing

The additional samples of 32 pigs were tested for the presence of the duplication causal for VWD. For this purpose, we used two pairs of primers. The first pair of primers flanks the duplication junction within the *VWF* gene and therefore results in a product of 215 bp only in case the duplication is present (VWD-affected and heterozygous pigs). In wild-type pigs, no product is produced. The second pair of primers is located within the *PROCR* (Protein C Receptor) gene and provides a PCR control, as a product of 377 bp is produced independently of the pigs’ genotype for VWD. Primers are specified in Table S1. All primers were applied to a multiplex PCR reaction using the *Taq* PCR Master Mix Kit (Qiagen, Hilden, Germany). This test was suitable to distinguish wild-type pigs (control band at 377 bp only) from those with the causal duplication in either homozygous or heterozygous status (bands for duplication and control at 215 and 377 bp, respectively). This test was exemplified in Figure S1.

To differentiate heterozygous from homozygous pigs, quantitative real-time (qRT) PCR on genomic DNA was tested for suitability. For this purpose, we used a LightCycler 1.0 (Roche Diagnostics Deutschland GmbH, Mannheim, Germany) and the SYBR Select Master Mix (Life Technologies GmbH, Darmstadt, Germany) according to the manufacturer’s instructions. We used the same two pairs of primers as described above. Data were analyzed using the ΔΔCT method (Livak and Schmittgen 2001). The amount of PCR product generated by the primers within *PROCR* was used to normalize the amount of product of the genomic duplication junction within *VWF* (ΔCT). The relative level of duplicated sequence was calculated using the average ΔCT of the 12 pigs homozygous for VWD as a calibrator to generate the ΔΔCT for all individuals and the level of duplicated product was subsequently calculated by 2^−ΔΔCT^. All assays were performed in duplicates.

### Data availability

Sequence data of this study are available at GenBank. Accession numbers were assigned for the complete coding sequences of a wild-type pig (MG372111) and a pig affected by VWD (MG372110) and for genomic sequences flanking the break points (KY073132, KY073133) and the duplication junction (KY073134), as well as for the sequence of the duplication junction on genomic DNA obtained by next generation sequencing (MG372112).

File S1 contains the sequences of break points and the duplication junction (KY073132, KY073133, KY073134) with further description. Table S1 contains all primers designed for this study. Table S2 and Table S3 contain all variants and genotypes detected in the sequenced pigs within the *VWF* cDNA and within the sequences spanning both duplication break points, respectively. Figure S1 provides an example of the genetic test, which is suitable to distinguish VWD-affected or heterozygous pigs from wild-type individuals.

## Results

### Sanger sequencing of cDNA

We analyzed the complete *VWF* coding sequence based on cDNA of one VWD-affected, one VWD-carrier, and one VWD-unaffected animal. The sequences included 58 bp of the 5′ untranslated region (UTR) and 94 bp of the 3′ UTR.

**Figure 1:**
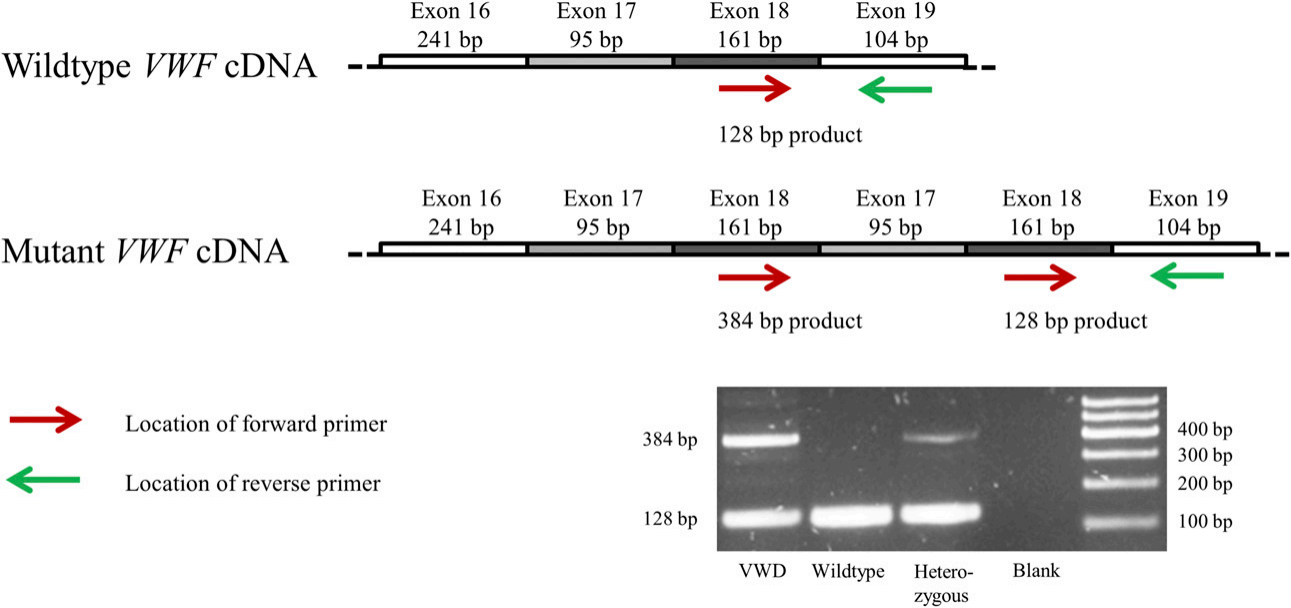
Illustration of wild-type and mutant *VWF* cDNA with location of the forward and the reverse primer and their band pattern on agarose gel following PCR. The band patterns of each one VWD-affected, wild-type, and heterozygous pig are shown. In the pig affected by VWD, two distinct bands are visible, one at the expected size of 128 bp and a larger one at 384 bp. The size difference of 256 bp corresponds with the combined size of exons 17 and 18 and results from the presence of the causal duplication including the two exons. In the wild-type pig, only a band at 128 bp is present and in the heterozygous individual the larger band is present, but much fainter.

**Figure 2:**
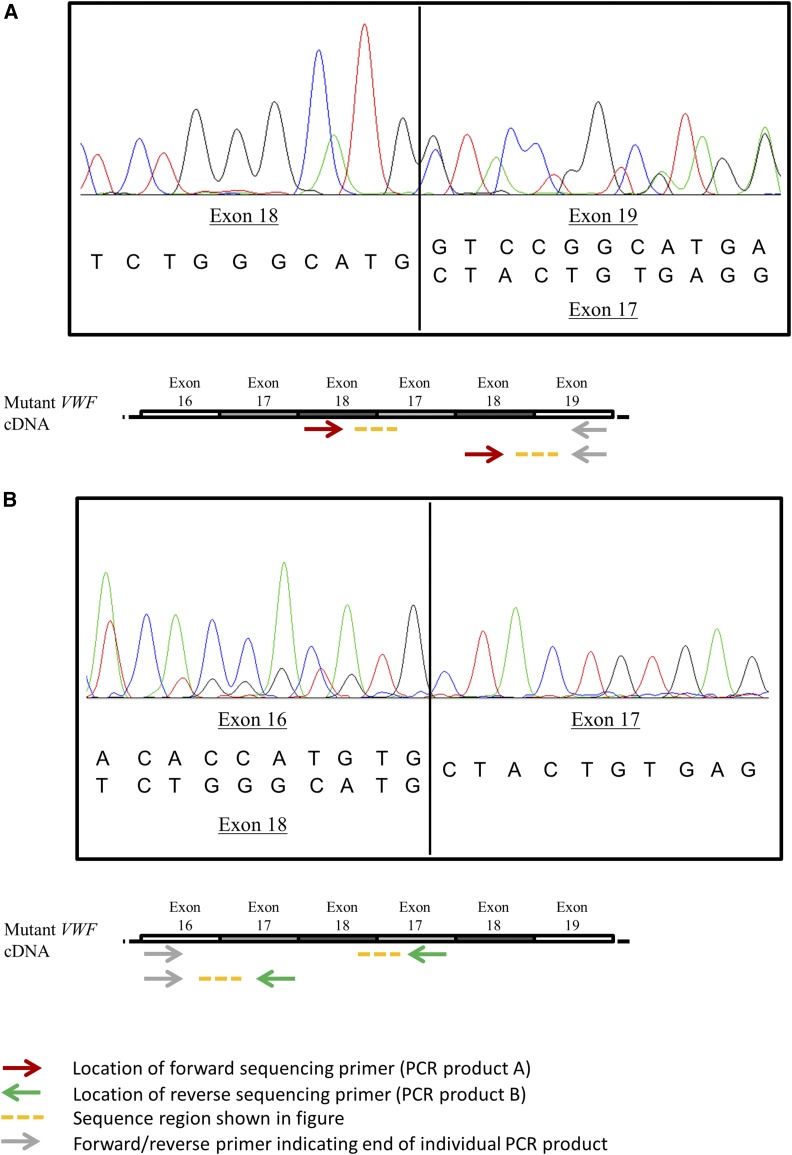
Sequences indicating the duplication. (A) Section of forward sequence on a PCR product spanning from exon 18 to exon 19 in the pig affected by VWD. Downstream of exon 18, sequences of exon 19 and exon 17 simultaneously start. (B) Section of reverse sequence on a PCR product spanning from exon 16 to exon 18 in the pig affected by VWD. Upstream of exon 17, sequences of exon 16 and exon 18 endings superpose each other.

In both animals homozygous or heterozygous for VWD, two PCR products were obtained for primer pairs spanning from exon 16 to exon 18, from exon 18 to exon 24, and from exon 18 to exon 19 (primers F, G, and R; Table S1), with one of the products located at the expected size and the second one being ∼250 bp larger. In the homozygous pig, the larger product was almost as strong as the expected product, while it was much fainter in the heterozygous pig (Figure 1). Sequencing of these products in the VWD-affected pig revealed sequence overlay starting at the borders of exon 18–19 in the forward as well as exon 17–16 in the reverse sequences. Evaluating the forward sequences, downstream of exon 18 the sequences of exon 19 and 17 started simultaneously (Figure 2A). In the reverse sequences, sequences of exon 16 and exon 18 likewise ended at the beginning of exon 17 (Figure 2B). Exons 17 and 18 combined are 256 bp in size, which corresponds with the size difference of the two PCR products described above. Therefore, we hypothesized the presence of a tandem duplication including exons 17 and 18. The presence of this two-exon duplication causes a frame shift downstream of exon 18 and a premature stop codon (p.Val814LeufsTer3).

### Next generation sequencing of duplication junction

To analyze the genomic background of this duplication, we performed next generation sequencing using long-range primers located within exon 18 (forward) and exon 17 (reverse). A fragment of ∼10 kb in size was amplified in heterozygous and VWD-affected pigs. As expected, no product was obtained in wild-type animals. The resulting sequence assemblies contained the duplication junction, which led to the conclusion that the sequence extending from 67,050,304 bp (intron 16) to 67,062,561 bp (intron 18) on *SSC5* (*Sscrofa10.2*) was tandemwise duplicated. The sequence was submitted to GenBank and assigned the accession number MG372112.

### Location of duplication break points on genomic DNA

**Figure 3:**
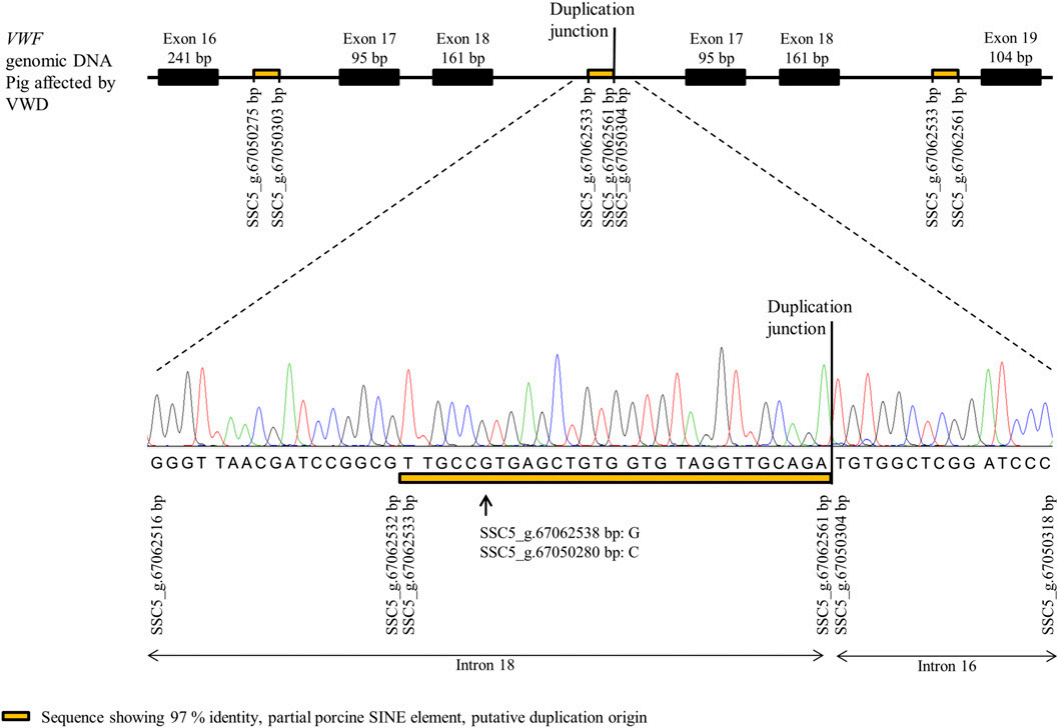
Sequence displaying the location of the duplication junction on genomic DNA of a pig affected by VWD type 3. Upstream of the duplication junction, a 29 bp sequence is indicated, which is part of a porcine SINE element and occurs with 97% identity from *SSC5_67*,*062*,*533* to *SSC5_67*,*062*,*561* bp and from *SSC5_67*,*050*,*275* to *SSC5_67*,*050*,*303* bp. The one diverging base is indicated and may lead to the conclusion that the actual location of this strand is *SSC5_67*,*062*,*533* to *SSC5_67*,*062*,*561* bp.

As the reference sequence was incomplete around the downstream duplication break point at *SSC5_67*,*062*,*561* bp, genomic regions around both previously determined break points as well as of the duplication junction were additionally Sanger-sequenced using the primers S and T (Table S1) and assigned GenBank accession numbers KY073132-4. As incidental findings, sequences of the duplication break point within intron 18 in our pigs contained another, small duplication of 194 bp (File S1) and some further deviations from the reference sequence (Table S2) without causal relevance for VWD. The region flanking the duplication break point at *SSC5_67*,*050*,*304* bp within intron 16 in our pigs included one variant (Table S2) and otherwise matched the reference sequence. The position of the duplication junction was confirmed (Figure 3 and File S1). The resulting duplication size based on the reference sequence complemented by the sequences of this study was 12,334 bp.

### Putative duplication origin

Subsequently, we analyzed our sequences for the characteristics and identity of the intron 16 and intron 18 duplication break points. A scan for repetitive elements showed the presence of porcine SINE elements with a total identity of 89% spanning both locations. An almost identical sequence of 29 bp directly precedes both break points (Figure 3). It shows an identity of 97%.

### Further analyses and variants detected

In the pig genome (*Sscrofa10.2*), *VWF* is located on chromosome 5 between 66,999,793 bp and 67,078,133 bp, spanning ∼78 kb of genomic sequence. Alignment of this porcine genomic DNA and the mRNA (NCBI accession number: NM_001246221.1) shows that the porcine gene consists of 52 exons. The open reading frame is 8424 bp, yielding a protein of 2807 amino acids (aa) plus the stop codon. Protein identity between human (NP_000543.2) and pig (NP_001233150.1) VWF is at 84%.

In addition to the 12.3 kb duplication, 60 additional variants were detected (Table S3). Of those, 40 were variable among the analyzed animals and 20 seem to be family-specific variants, identified by comparison to the reference sequence. Three of the variable and five of the family-specific variants were nonsynonymous.

### Immunohistochemistry

**Figure 4:**
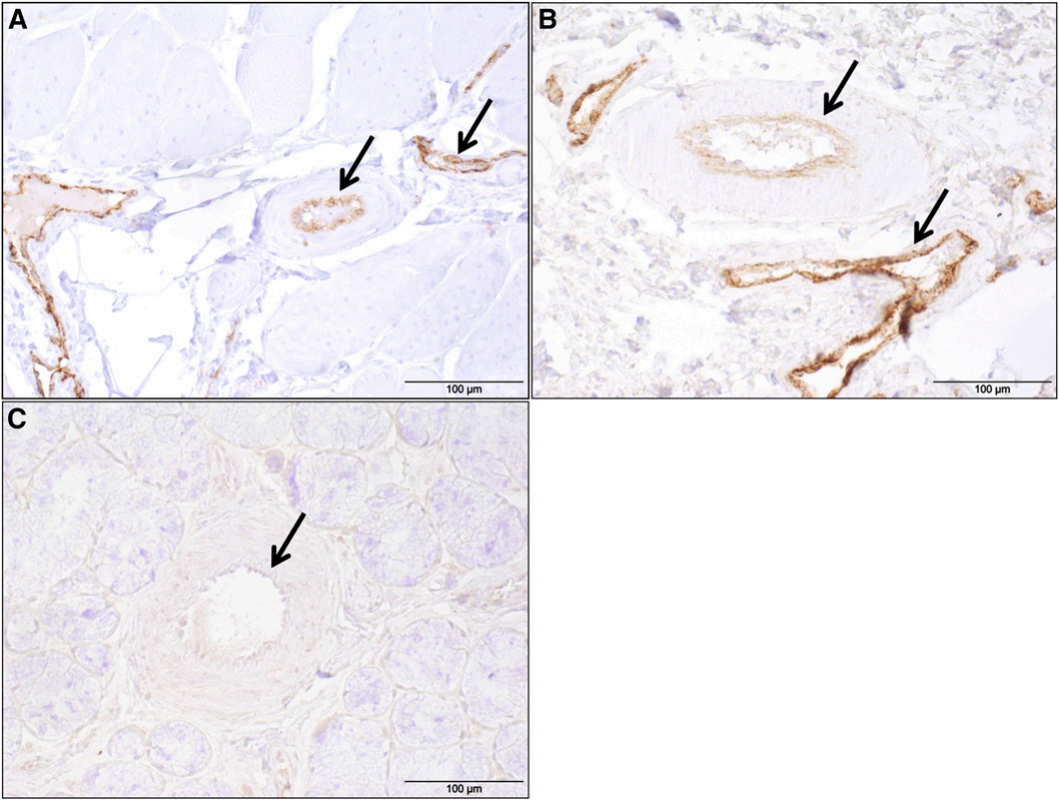
Immunohistochemistry of Von Willebrand factor (VWF) in porcine esophagus. Light microscopic images of porcine esophagus showing representative immunohistochemical staining for VWF. The endothelial cells (arrows) of blood vessels from both the wild-type (A) and the heterozygous pig (B) show intense staining. In contrast, the vascular endothelial cells of the VWD-affected animal (C) show very weak to no staining. Scale bars = 100 μm.

The staining intensity for VWF distinctly differed between the tissue of the pig affected by VWD compared to those of the wild-type and heterozygous pigs. The endothelial cells of the VWD-affected animal showed no or weak staining (Figure 4C). In contrast, the endothelial cells of the wild-type and the heterozygous animals showed intense staining (Figure 4, A and B). The staining intensity reflects the presence of VWF protein.

### Evaluation of nonsense-mediated decay (NMD)

**Figure 5:**
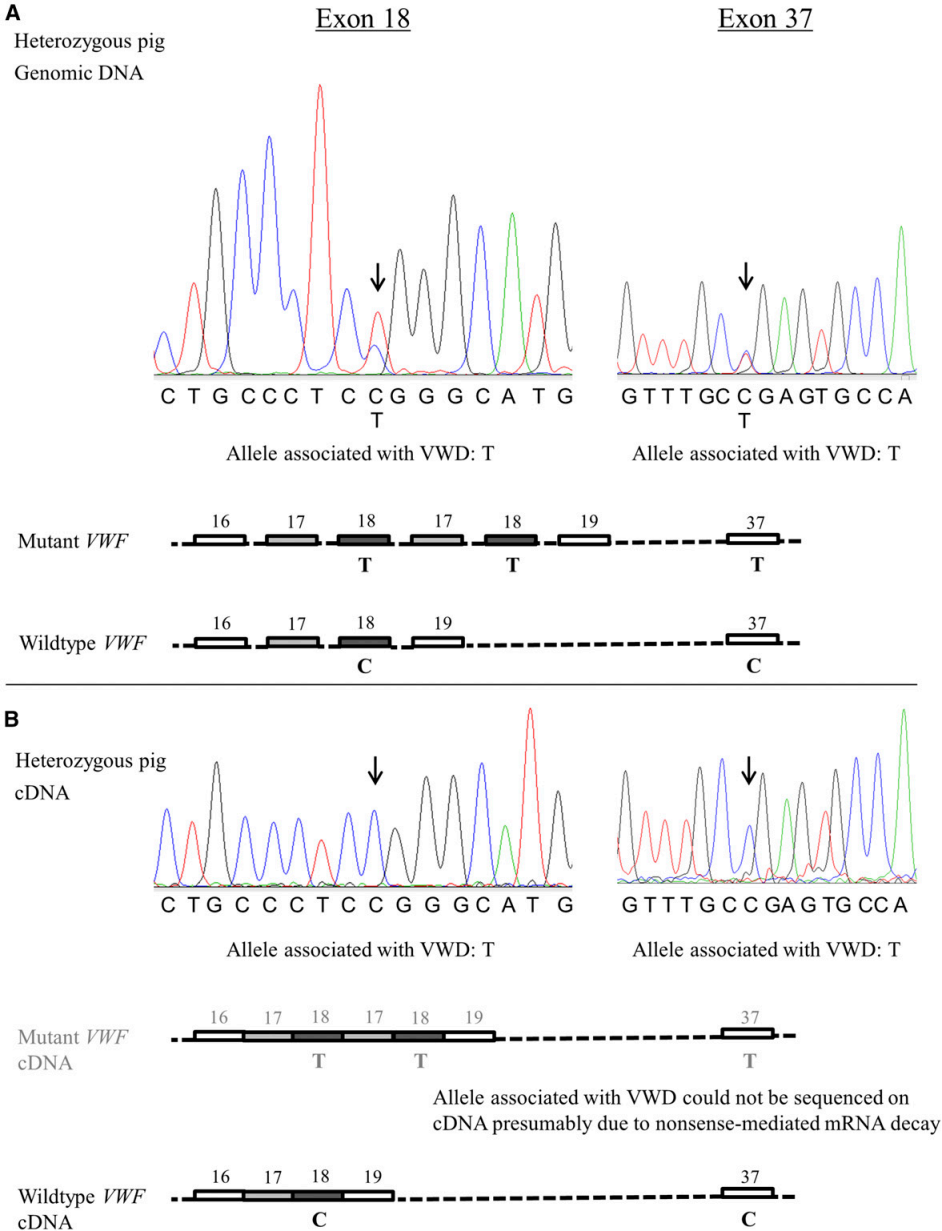
Comparative sequencing of variants on cDNA and genomic DNA suggests presence of NMD. Variants located within exon 18 (included in duplication) and exon 37 (not included in duplication) comparatively sequenced on genomic DNA and cDNA of a pig heterozygous for VWD type 3. (A) On genomic DNA, both alleles of each variant are clearly visible. In addition, for the variant located within exon 18 and thus within the VWD duplicated region, the allele associated with VWD shows the expected chromatogram-peak of about double the height of the allele associated with the wild type. (B) On cDNA, however, the allele associated with VWD does not show up. These differences between sequences of cDNA and genomic DNA can be explained by NMD of mRNAs containing the causal duplication.

For 24 of the variable genetic changes detected within *VWF* cDNA, the pig heterozygous for VWD type 3 was homozygous for the allele associated with the wild type. As mRNA containing a premature termination codon may be subject to NMD, two sequence variants located within exon 18 (included in duplication) and two within exon 37 (not part of the duplication) were comparatively sequenced on genomic DNA and cDNA of the five pigs. For both heterozygous pigs, the sequences obtained on genomic DNA showed the heterozygous genotype for all four variants. On cDNA, however, the alleles associated with VWD did not show up. In addition, for the variants located within the genomic region duplicated in VWD, the allele associated with VWD showed the expected chromatogram-peak of about double height of the allele associated with the wild-type (Figure 5).

### Genetic diagnosis in additional pigs

Of the additional 32 pigs, all 24 of the pigs phenotypically VWD-carriers or VWD-affected were also tested positive for the causal 12.3 kb duplication by PCR. The eight remaining pigs classified as VWD-unaffected were tested negative for the duplication.

For the differentiation of heterozygous from VWD-affected pigs, qRT PCR on genomic DNA was tested for suitability. The amount of product generated of the duplication junction site was evaluated relative to an independent PCR product. On average, the 12 VWD-carriers showed 51.7 ± 12.1% of the amount of duplication product of the 12 pigs affected by VWD (101.1 ± 16.0%). The product levels ranged between 79.7 and 138.5% in the pigs affected by VWD and between 38.4 and 78.7% in heterozygous pigs and were significant at p-value < 0.0001 using the Student’s *t*-test.

## Discussion

In this study we describe a duplication associated with VWD type 3 in pigs. The clinical signs of this disease in pigs match those in humans to a large extent and is why affected pigs provide a reliable animal model for the disease (Nichols *et al.* 2010). Though Bahou *et al.* (1988) performed linkage analyses which indicated the location of the molecular defect for VWD within or near to the *VWF* gene, the causal genetic variant remained undetected. Analyzing *VWF* cDNA and genomic DNA of VWD-affected, VWD-carriers, and VWD-unaffected pigs, we specified the putatively causal variant as a 12.3-kb genomic tandem duplication including the exons 17 and 18. This duplication causes a frame shift with a premature stop codon three amino acids downstream of the end of exon 18 (p.Val814LeufsTer3).

VWF is a glycoprotein synthesized in vascular endothelial cells as well as megakaryocytes of the bone marrow, consisting of in total 2813 aa in human and 2807 aa in pigs. Human VWF consists of a 22-aa signal peptide and a 741-aa propeptide, which are encoded by the first 17 exons and a small part of exon 18, and a mature protein of 2050 aa encoded by exons 18–52 (Sadler 1998; Lillicrap 2005). Sequence alignment of human and porcine sequences and proteins indicates a signal peptide of 22 aa, a propeptide of 740 aa, and a mature peptide of 2045 aa in pigs, with similar affiliation of exons as in humans. Therefore, the premature stop codon created by the duplication described in this study causes a severely truncated mature VWF peptide of 51 aa with 1994 aa missing. Thus, all functionally important domains, as for example FVIII- and collagen-binding as well as di- and multimerization domains (Ng *et al.* 2015), are absent from this truncated mature VWF peptide.

The duplication was present in all VWD-affected and VWD-carrier pigs and absent from all VWD-unaffected pigs analyzed. Though the sample size is small, the protein-truncating variant type as well as its severe functional effect on the VWF protein supports the causality of the duplication for VWD.

In human VWD type 3, homozygous or compound heterozygous premature termination of the protein is the major genetic cause as well. Two of those variants were detected in exons 17 and 18, respectively. These are a 2-bp deletion (p.Leu757ValfsTer22; Baronciani *et al.* 2003) and a 1-bp deletion (p.Pro812ArgfsTer31; Zhang *et al.* 1992), each causing a frameshift and a premature termination codon. But even truncations occurring as far downstream as in exon 45 may cause VWD of this severe type (ISTH-SSC VWF Database, http://www.ragtimedesign.com/vwf/). In addition, all genetically resolved cases of VWD type 3 in dogs are caused by premature termination of the VWF protein (Rieger *et al.* 1998; Venta *et al.* 1998, 2000). Of the 589 reported, protein-truncating variants within human *VWF*, 572 were associated with VWD type 3 and only 17 with other types of the disease (types 1, 2A, or 2N; ISTH-SSC VWF Database). Though one duplication of 45 bases within *VWF* exon 37 causing VWD type 3 has been reported in humans (Kasatkar *et al.* 2014), to our knowledge no duplication causing a premature termination of *VWF* has been described before in any species.

Large tandem duplications including gene sections or even complete genes are known to frequently result in genetic diseases. In human, for example, a tandem duplication of five exons of the *ALL1* gene is associated with acute myeloid leukemia (Strout *et al.* 1998). In animals, large duplications have been reported to be causative for certain phenotypes. A 4.6-kb duplication within intron 6 of *STX17* was shown to cause gray coat color in horses (Rosengren Pielberg *et al.* 2008), and an even larger duplication of 133 kb including the genes *FGF3*, *FGF4*, *FGF19*, and *ORAOV1* causes a dorsal hair ridge and predisposition for dermoid sinuses in dogs (Salmon Hillbertz *et al.* 2007).

The individual mechanism of duplication formation can reliably be analyzed by examining the duplication junction (Hu and Worton 1992). This method was also implemented in the present study. Both break points of the duplication are located within porcine SINE elements at sequence sections which show an identity of 97% among each other. Duplications often originate from homologous recombination involving SINE elements (Hu and Worton 1992). In primates, SINE elements of Alu type are known to promote unequal homologous recombination events. When occurring intragenically, they frequently cause deletion or duplication of exons (Deininger and Batzer 1999). Therefore, the porcine SINE elements detected at the duplication break points may be considered the putative origin of the tandem duplication causal for VWD in pigs.

A differentiation of wild-type pigs from those heterozygous or homozygous for the 12.3-kb duplication can be easily and reliably performed by PCR. A further differentiation of heterozygous and VWD-affected genotypes could not be achieved by simple PCR due to the large duplication size. However, qRT PCR on genomic DNA seems to be a promising approach. The average relative level of PCR product of the duplication junction is expected to be at 100% in VWD-affected and 50% in heterozygous pigs, which corresponds well to the values measured in this study. However, though the ranges of these levels did not overlap for both genotypes, the maximum level detected in the heterozygous pigs (78.7%) was close to the minimum level in the pigs affected by VWD (79.7%), and the spread between maximum and minimum level was 58.8% in affected and 40.3% in heterozygous pigs. Therefore, a sufficient number of individuals with known genotype is required to determine the calibrator needed for this test. However, as VWD type 3 affected individuals are usually diagnosed early in life, the typical application of a genetic test regarding this disease will be the discrimination of heterozygous from wild-type pigs. As values of the VWF:AG between 50 and 80% cannot be safely assigned a specific VWD type 3 phenotype, because VWD-carriers and VWD-unaffected individuals may both show values within this range (Goodeve and James 2014; Gadisseur *et al.* 2013), our genetic test is especially beneficial to differentiate those pigs.

*VWF* contains a variety of important domains, including binding sites for factor VIII, collagen, and platelets, as well as sites for di- and multimerization, storage, cleavage, and others. Nevertheless, benign variants are a common and frequent finding even within these domains in humans (Flood 2014). Hundreds of exonic or closely flanking intronic variants have been reported, including 80 nonsynonymous variants, which did not impair the gene function (Goodeve and James 2014). This seems to be similar in pigs, as 60 genetic variants not associated with VWD were detected in our porcine sequences.

qRT PCR and cDNA sequencing revealed a greatly reduced level of the mutant transcripts compared to the wild-type transcripts in heterozygous pigs. This is probably due to NMD, a mechanism that degrades mRNAs containing a premature termination codon located at least 50 nucleotides upstream of the final exon–exon junction (Hug *et al.* 2016), which applies for *VWF* mRNA containing the duplication causal for VWD. Furthermore, as the premature stop codon caused by the duplication is located downstream of the sequence coding for the propeptide and mainly truncates the sequence coding for the VWF mature peptide, it could be assumed that the propeptide creation should be unaffected. However, free VWF propeptide in the plasma was below the limits of detection in VWD-affected pigs according to a study of Turecek *et al.* (1999). As NMD leads to a quick decomposition of mRNA, which is the template for protein translation, all parts of VWF protein are reduced. The presence of NMD is further supported by the results of our immunohistochemistry analysis. While expression of VWF protein was obvious in the endothelium of wild-type and heterozygous pigs, almost no VWF protein was detectable in the pig affected by VWD type 3, though polyclonal antibodies were used.

In conclusion, a genomic 12.3-kb tandem duplication including exons 17 and 18 of *VWF* is the putative cause for VWD in pigs by inducing a frameshift followed by a premature stop codon (p.Val814LeufsTer3). Differentiation of wild-type pigs from those heterozygous for VWD can now be performed by a rapid and simple genetic analysis.

## Supplementary Material

Supplemental material is available online at www.g3journal.org/lookup/suppl/doi:10.1534/g3.117.300432/-/DC1.
