# Supplementary figures and images for: A 12.3-kb Duplication Within the VWF Gene in Pigs Affected by Von Willebrand Disease Type 3
Source: G3 (Bethesda). 2017 Dec 5;8(2):577–85. doi: 10.1534/g3.117.300432 (PMC5919753; doi:10.1534/g3.117.300432)

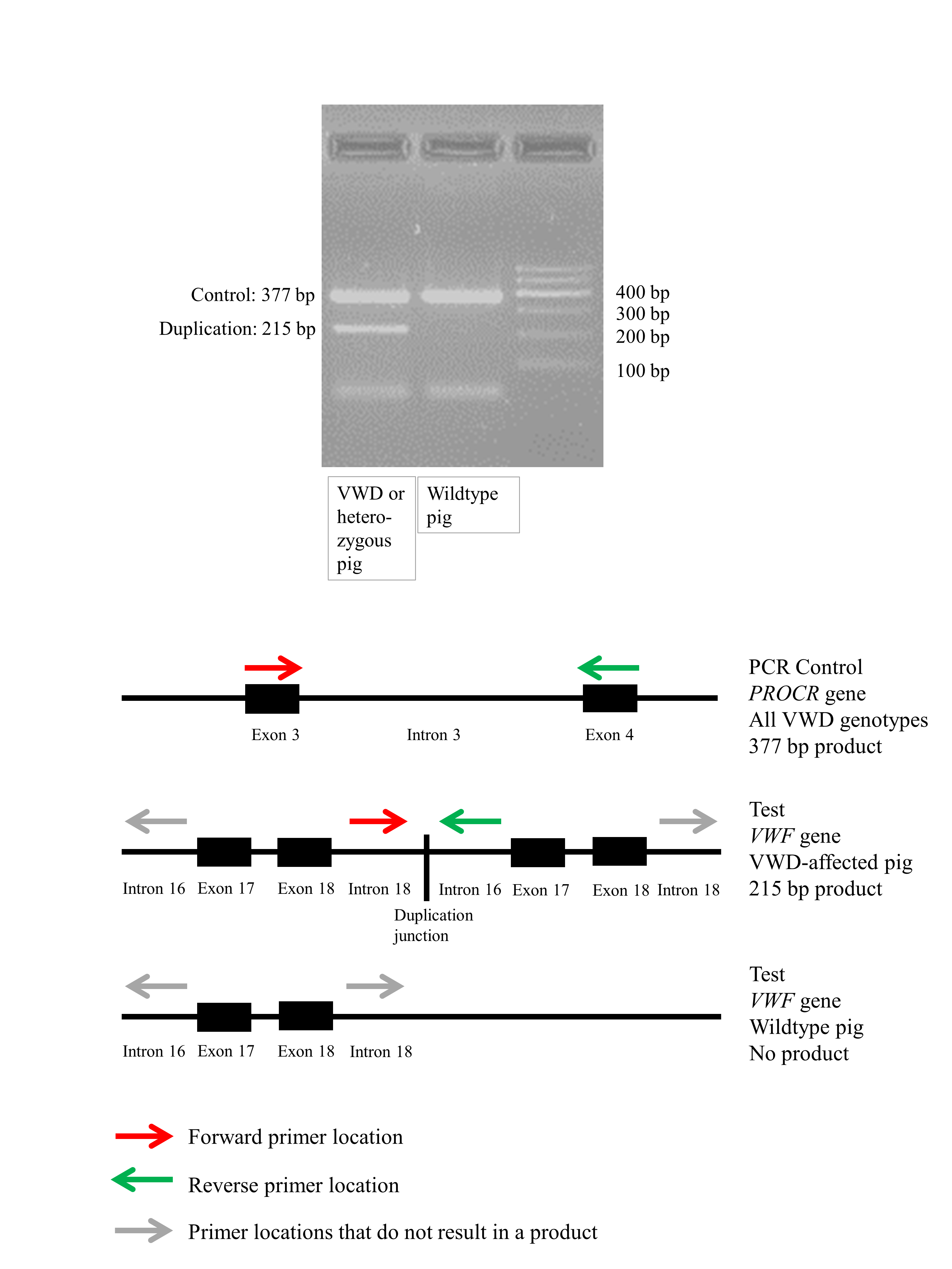

Supplement: Supplementary file 1 [file 577FigureS1.tif]
