# Supplementary material for: A 12.3-kb Duplication Within the VWF Gene in Pigs Affected by Von Willebrand Disease Type 3
Source: G3 (Bethesda). 2017 Dec 5;8(2):577–85. doi: 10.1534/g3.117.300432 (PMC5919753; doi:10.1534/g3.117.300432)
Supplement: Supplementary file 5 [file 577FileS1.pdf]

File S1. Genomic sequences flanking the upstream and downstream break points, respectively, as well as the junction of the duplication causal for VWD. The duplication break points are located within *VWF* intron 16 (1.) and intron 18 (2.). At the duplication junction (3.), which is only present in VWD-affected or heterozygous pigs, the sequence of intron 18 is terminated at the downstream break point and continues from the upstream break point within intron 16.

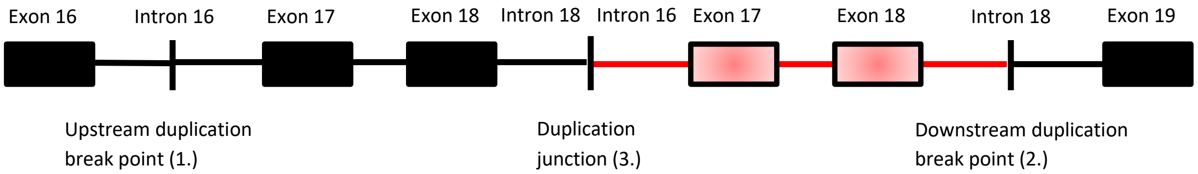

1.) Partial genomic sequence of *VWF* intron 16 in wildtype and VWD-affected pigs. This sequence flanks the upstream break point of the duplication causal for VWD (GenBank accession number: KY073132; Sscrofa10.2_SSC5_67050039-67050375 bp)

GAGGGTATTTTTAAAGCAGATGTTAGTTAAAAACCAGAAGGCAAGGACCTGCATT
TGCCCAGAGAGGGAAAGGGCTGATTCCATTCTTCATTCAATGAAAGTTGATGGAA
TAAATGGCACATTAAAAATGGTCAAAGTCGGAGTTCCCGTCATAGCTCAGCAGAA
CGAATTTGACTTGGAACCATGAGGTTGTGGGTTCGATCCCTGGCCTCGCTCAGTG
GGTTAAGGATCCGGCCTTGCC**C**TGAGCTGTGGTGTAGGTTGCAGA [Upstream duplication break point]
TGTGGCTCGGATCCCATGTTGCTGTGGCTGTGGTGTAGGCCGGTGGCTACAGCTC
AGATTATGCCGTGGGTG

2.) Partial genomic sequence of *VWF* intron 18 in wildtype and VWD-affected pigs. This sequence flanks the downstream break point of the duplication causal for VWD (GenBank accession number: KY073133; Sscrofa10.2_SSC5_67062283-67062669 bp)

CAGGAACTCCTGCTGCTGATTTTTAAAACAGAGGAGGAGTTCCCATCGTGGCGTA
GTGGTTAACGAATCCGACTAGGAACCATGAGGTTGCGGGTTCGATCCCTGCCCTT
GCTCAGTGGGTTAACGATCCGGCGTTGCC**G**TGAGCTGTGGTGTAGGTTGCAGACG
CGGCTCGGATCCCGCGTTGCTGTGGCTCTGGTGTGGGCTGGCAGCTACAGCTCCA
ATCAACCCCTAAGTTCCCATCGTGGCGTAGTGGTTAACGAATCCGACTAGGAACC
ATGAGGTTGCGGGTTCGATCCCTGCCCTTGCTCAGTGGGTTAACGATCCGGCGTT
GCC**G**TGAGCTGTGGTGTAGGTTGCAGA [Downstream duplication break point]

CGCGGCTCGGATCCCGCGTTGCTGTGGCTCTGGTGTGGGCTGGCAGCTACAGCTC
CAATCAACCCCTAACCTGGGAAACTCCGTATGCCGTGGGAGTGGCCCTAGAA

3.) Genomic sequence flanking the duplication junction in VWD-affected (or heterozygous) pigs. At this junction the sequence of *VWF* intron 18 is terminated at the downstream break point (2.) and continued from the upstream break point (1.) within intron 16 (GenBank accession number: KY073134)

AGGAACTCCTGCTGCTGATTTTTAAAACAGAGGAGGAGTTCCCATCGTGGCGTAG
TGGTTAACGAATCCGACTAGGAACCATGAGGTTGCGGGTTCGATCCCTGCCCTTG
CTCAGTGGGTTAACGATCCGGCGTTGCC**G**TGAGCTGTGGTGTAGGTTGCAGACGC
GGCTCGGATCCCGCGTTGCTGTGGCTCTGGTGTGGGCTGGCAGCTACAGCTCCAA
TCAACCCCTAAGTTCCCATCGTGGCGTAGTGGTTAACGAATCCGACTAGGAACCA
TGAGGTTGCGGGTTCGATCCCTGCCCTTGCTCAGTGGGTTAACGATCCGGCGTTG
CC**G**TGAGCTGTGGTGTAGGTTGCAGA [Duplication_junction]
TGTGGCTCGGATCCCATGTTGCTGTGGCTGTGGTGTAGGCCGGTGGCTACAGCTC
AGATTTGCCGTGGGTG

Comments:

Sequences marked in yellow and turquois indicate both parts of another small duplication within the sequence including the downstream duplication break point. This duplication was incidently revealed by sequencing what was marked by a stretch of [N] within the reference sequence. It was present in samples of all our pigs and is not associated with VWD.

Red letters indicate a sequence, which occurs almost identically within the sequences including the upstream and the downstream break point, respectively (the bold letter indicates the only difference). This sequence probably was the primary cause for the development of the duplication causal for VWD.

Putative break points and causal duplication junction are provided in square bracket within each sequence.

Differences between the reference sequence and our sequences, as well as variation among the sequenced pigs are provided within Table S2 and Table S3.
